# Longitudinal changes in minerals are influenced by immunosuppressive treatment in men with granuloma disease

**DOI:** 10.1007/s40618-025-02607-3

**Published:** 2025-05-12

**Authors:** Sam Kafai Yahyavi, Ida Enggaard Kaae, Anders Juul, Ebbe Eldrup, Martin Blomberg Jensen

**Affiliations:** 1 Division of Translational Endocrinology, Department of Endocrinology and Internal Medicine, Copenhagen University Hospital - Herlev and Gentofte, Copenhagen, Denmark; 2 Group of Skeletal, Mineral and Gonadal Endocrinology, Department of Growth and Reproduction, Copenhagen University Hospital– Rigshospitalet, Copenhagen, Denmark; 3 Department of Growth and Reproduction, Copenhagen University Hospital– Rigshospitalet, Copenhagen, Denmark; 4 International Centre for Research and Research Training in Endocrine Disruption of Male Reproduction and Child Health (EDMaRC), Copenhagen University Hospital– Rigshospitalet, Copenhagen, Denmark; 5 Department of Clinical Medicine, Copenhagen University Hospital, Copenhagen, Denmark; 6 Department of Endocrinology, Herlev-Gentofte University Hospital, Copenhagen, Denmark

**Keywords:** Cosmetic oil injections, Granuloma, Phosphate, Minerals, Magnesium, Electrolytes

## Abstract

**Purpose:**

To investigate whether granuloma formation following self-administered cosmetic oil injections affects mineral homeostasis, specifically calcium, magnesium, phosphate, iron, sodium, and potassium, and to assess the potential impact of prednisolone treatment on these mineral levels.

**Methods:**

In this retrospective study, we reviewed blood samples from baseline through a follow-up period of 48 months in patients referred to a tertiary center at Herlev Hospital, Denmark. Changes in serum minerals over time were assessed by a linear mixed model for repeated measures.

**Results:**

A total of 111 patients were included. Men who injected > 2,000 mL paraffin oil had higher total and ionized calcium (*p* = 0.029 and *p* < 0.001), lower PTH (*p* < 0.001), but also lower magnesium (*p* < 0.001) and higher sodium (*p* = 0.048) compared to those who had injected < 500 mL. Men with manifest hypercalcemia at baseline (*n* = 32) compared to men with normocalcemia (*n* = 79) experienced an increase in serum PTH and phosphate concentrations over time (*p* = 0.042 at 48 months), and also a transient increase in iron concentration, although this reached baseline levels again after 24 months. Prednisolone lowered calcium in hypercalcemic men but also decreased serum magnesium (*p* = 0.027 after 36 months), phosphate, and increased serum iron concentration.

**Conclusion:**

Men who had injected large volumes of paraffin oil were more likely to have hypercalcemia, lower magnesium, and higher sodium concentrations. Minor aberration in serum minerals was more frequent in patients with more pronounced disease and this may likely be a poor prognostic sign although the mechanism behind this observation remains unclear.

## Introduction

Some individuals inject oils into their muscles for aesthetic reasons and this may initially help to obtain the desired cosmetic results but can lead to chronic inflammatory reactions and granuloma formation [1–3]. The adverse response poses a substantial clinical challenge, often resulting in hypercalcemia, disfiguring, and painful complications that require complex management strategies including medical treatment with corticosteroids [4–8]. Understanding the underlying disease mechanisms caused by the granuloma formation is in this context essential to improving the therapeutic options. The exact prevalence of granulomas following injections remains unclear, as most available data derive from cohorts enriched for patients experiencing complications. Over the years, the physiology of how granulomas cause hypercalcemia some years after oil injections has been well described [9]. In brief, paraffin oil injections can lead to granulomas that are rich in macrophages and lymphocytes and these cells can activate vitamin D [10, 11]. Under normal circumstances, calcium homeostasis is tightly regulated by active vitamin D through intestinal absorption, renal excretion, and skeletal release to maintain stable serum concentrations [12, 13]. Vitamin D undergoes hepatic and renal conversions to its active form, 1,25-dihydroxyvitamin D, which promotes calcium and phosphate absorption and reabsorption [14, 15]. In granulomas, the activating enzyme CYP27B1 is induced by cytokines, and the inactivating enzyme CYP24 A1 is not upregulated, causing high extra-renal production of 1,25-dihydroxyvitamin D and thereby elevated serum calcium levels [16].

The homeostasis of serum minerals plays an essential role in maintaining cellular function and overall health. Hypercalcemia can cause symptoms such as nausea and dizziness, while more advanced hypercalcemia can lead to significant complications, including cognitive impairment, seizures, cardiac arrhythmias, renal failure, and in some cases fatal [17, 18]. Other minerals besides calcium are also integral to numerous physiological functions. Iron is a critical component of hemoglobin and vital for oxygen transport and energy production in cells [19, 20]. Phosphate is crucial for energy production DNA and RNA synthesis, and bone mineralization [21, 22], and magnesium is important for muscle and nerve function and protein synthesis [23, 24]. Sodium and potassium are essential for maintaining fluid balance, and muscle and nerve function [25].

While the focus in granuloma disease extensively has been on calcium homeostasis [9], the changes and effects of other minerals have not been characterized. Understanding the role of these factors in granuloma disease and during treatment with immunosuppressants could give new insights into the pathophysiology of the condition and potentially lead to better monitoring and treatment. For example, disturbances in iron metabolism may affect erythropoiesis and immune responses, while phosphate imbalance can impact energy metabolism and calcification, and magnesium levels can influence PTH and immune function and thereby inflammation. Similarly, imbalances in sodium and potassium might influence neuromuscular function and fluid balance, which are all relevant in the context of granuloma disease.

In this study, we assessed serum levels of calcium, sodium, potassium, magnesium, phosphate, and iron during at first visit and up to 48 months of follow-up to show the mineral-related biochemical changes occurring in patients with granulomatous disease after cosmetic oil injection. Currently, there are no studies that have evaluated the changes over time, and by analyzing these minerals, we aim to deepen the understanding of the pathophysiology of granuloma disease and to identify potential biomarkers for monitoring disease progression and therapeutic efficacy.

## Materials and methods

### Study setting

**Fig. 1:**
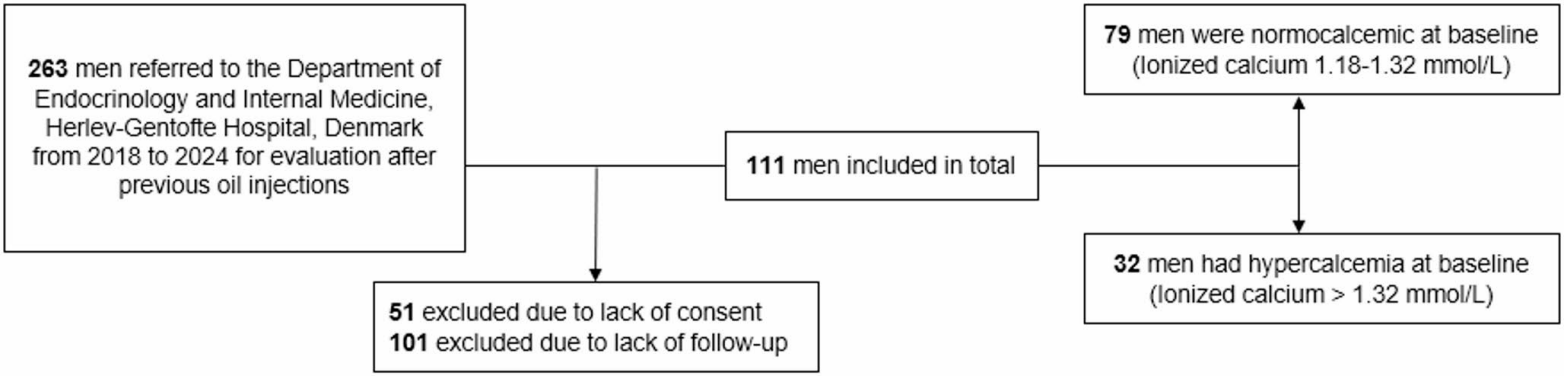
Selection of the study population. Flowchart of selection of study population including exclusions

Since 2018, patients with previous paraffin oil injections have been referred to the Department of Endocrinology at Herlev-Gentofte University Hospital (HGH), Copenhagen, Denmark for clinical evaluation and treatment. Patients are usually referred via either a physician because of hypercalcemia or pain from the injection site, or they self-refer. At the first visit, all patients are interviewed regarding the history (i.e., site, date, amount, and temperature) of cosmetic oil injections and on general lifestyle factors such as smoking, alcohol consumption, daily medication intake, as well as their history and current usage of anabolic steroids. Baseline characteristics of this cohort have previously been reported [26], and the effects of medical and surgical treatment have been published [9, 27]. Until June 2024, 263 patients have been referred to the department. 51 did not sign a consent form to participate in this study, and a total of 101 patients were excluded due to lack of follow-up or death during follow-up. A flow chart of the referrals and study cohort is shown in Fig. 1.

### Outcomes

For this retrospective study, we have gathered information from all the blood samples that have been drawn, and the main outcome will be changes in serum minerals, comprised of phosphate, calcium, magnesium, iron, potassium, and sodium. The secondary outcomes will be changes in regulators of the mineral homeostasis, i.e., serum concentrations of 25(OH)D, PTH, and 1,25(OH)_2_D_3,_ and are an extension of previous work [9, 27].

### Biochemistry

Blood samples were primarily drawn and analyzed at the Department of Clinical Biochemistry at Herlev-Gentofte University Hospital (HGH). Serum phosphate (coefficient of variation (CV) < 5%), serum total calcium (CV < 3%), serum PTH (CV < 5%), serum 25(OH)D (CV < 11%), serum magnesium (CV < 7%), serum sodium (CV < 2.5%), serum potassium (CV < 3%), and serum creatinine (CV < 4%) were measured using Siemens Atellica CH 930, whereas serum ionized calcium (CV < 4%) was measured on ABL 837. Some patients were referred from distant regions of Denmark and had their initial blood samples drawn at their local hospital or laboratory. GFR was determined by the CKD-EPI equation [28].

### Statistical analysis

Descriptive statistics are presented as means with standard deviation (SD) for continuous variables in Table 1, while categorical variables are presented as numbers with percentages. Significant differences between groups were evaluated with a crude comparison of means (t-test). Differences between oil injection groups in Fig. 1 were assessed by the one-way ANOVA test and post-hoc pairwise comparisons adjusted with Dunnett’s test. For the prednisolone subgroup in Fig. 4, a linear mixed model analysis was conducted to evaluate changes in mean serum levels of minerals over time, from baseline up to 48 months of follow-up [29, 30]. A significance level of *p* < 0.05 was considered statistically significant. All statistical calculations were conducted using the statistical software R, version 3.4.1 (R Core Team 2021. R: A Language and Environment for Statistical Computing. R Foundation for Statistical Computing, Vienna, Austria. http://www.R-project.org).

**Table 1:** Baseline characteristics of the study population. BMI: body mass index; PTH: parathyroid hormone; 25(OH)D: 25-hydroxyvitamin D; 1,25(OH)_2_D_3_: 1,25-dihydroxyvitamin D; eGFR: estimated glomerular filtration rate. Significance levels; p<0.05 after crude comparison of means (t-test)

	Reference interval	All men		Normocalcemic men		Hypercalcemic men		*P*-value
		*n*	Mean (SD)	*n*	Mean (SD)	*N*	Mean (SD)	
Age (years)		111	38.4 (7.9)	79	36.5 (7.8)	32	40.2 (8.0)	0.147
Height (cm)		111	180 (7)	79	180 (7)	32	180 (7)	0.654
Weight (kg)		111	92.5 (12.8)	79	90.6 (12.5)	32	94.3 (13.3)	0.578
BMI (kg/m²)		111	28.4 (3.4)	79	27.9 (3.6)	32	28.8 (3.3)	0.420
Smoking, no. (%)		111	55 (51)	79	36 (46)	32	19 (59)	0.188
Alcohol use, no. (%)		109	61 (57)	77	41 (53)	32	20 (63)	0.375
Volume oil injected (mL)		109	951 (876)	77	852 (822)	32	1189 (968)	0.091
Time since oil injection (years)		111	12.1 (3.6)	79	11.4 (3.3)	32	12.8 (3.9)	0.077
Total calcium (mmol/L)	(2.15–2.51)	109	2.45 (0.23)	77	2.36 (0.08)	32	2.69 (0.30)	–
Ionized calcium (mmol/L)	(1.18–1.32)	109	1.30 (0.14)	77	1.24 (0.04)	32	1.46 (0.18)	–
Magnesium (mmol/L)	(0.70–0.95)	106	0.80 (0.08)	76	0.81 (0.07)	30	0.79 (0.10)	0.446
Phosphate (mmol/L)	(0.81–1.45)	107	1.00 (0.26)	76	0.99 (0.18)	31	1.01 (0.41)	0.762
Sodium (mmol/L)	(135–145)	107	140.1 (2.2)	76	140.4 (2.1)	31	139.5 (2.4)	0.088
Potassium (mmol/L)	(3.6–4.6)	107	4.0 (0.3)	76	4.0 (0.2)	31	3.9 (0.3)	0.298
Iron (µmol/L)	(9–34)	107	18.4 (7.1)	76	19.2 (7.1)	31	16.3 (6.8)	**0.041**
Ferritin (µg/L)	(12–300)	107	363 (289)	76	346 (256)	31	403 (367)	0.127
Hemoglobin (mmol/L)	(8.3–10.5)	107	9.7 (0.9)	76	9.9 (0.8)	31	9.3 (1.0)	**0.012**
PTH (pmol/L)	(2.0–8.5)	109	2.2 (1.4)	77	2.6 (1.3)	32	1.0 (0.7)	**< 0.001**
25(OH)D (nmol/L)	(> 50)	108	48 (24)	76	46 (25)	32	54 (21)	0.081
1,25(OH)_2_D_3_ (pmol/L)	(60–160)	109	127 (52)	77	116 (41)	32	154 (66)	**0.005**
Albumin (g/L)	(36–48)	109	41 (4)	77	41 (4)	32	39 (4)	**0.014**
Creatinine (µmol/L)	(60–105)	109	94 (15)	77	85 (10)	32	115 (21)	**< 0.001**
eGFR (mL/min/1.73 m^2^)	(> 90)	109	84 (18)	77	91 (12)	32	69 (21)	**< 0.001**

BMI: body mass index; PTH: parathyroid hormone; 25(OH)D: 25-hydroxyvitamin D; 1,25(OH)_2_D_3_: 1,25-dihydroxyvitamin D; eGFR: estimated glomerular filtration rate. Significance levels; p<0.05 after crude comparison of means (t-test)

### Ethics

The data used in this study was collected in accordance with the second Helsinki Declaration. The study was approved by the Ethics Committee from the Region Capital of Denmark (journal no. H-19010297) and has been listed at clinicaltrial.gov (NCT04265599).

## Results

### Baseline characteristics

The study population consisted of 111 men with prior oil injections, where the severity of the disease was assessed by stratifying the men according to the degree of calcium imbalance: Men with normocalcemia, or men with manifest hypercalcemia (ionized calcium > 1.32 mmol/L and/or albumin corrected calcium > 2.51 mmol/L) (Table 1). The mean age of all men was 38.4 years (SD 7.9), with a mean BMI of 28.4 kg/m² (SD 3.4). There were no differences in the two groups in age (*p* = 0.147), height (*p* = 0.654), weight (*p* = 0.578), BMI (*p* = 0.420), smoking (0.188), or alcohol use (0.375). The men had injected an average volume of 951 mL (SD 876) of oil, with hypercalcemic men injecting more than men with normocalcemia (1189 mL vs. 852 mL, *p* = 0.091). The average time since oil injection was 12.1 years (SD 3.6) across all men, also slightly longer in hypercalcemic men than in normocalcemic men (12.8 years vs. 11.4 years, *p* = 0.077). As men were stratified according to calcium concentrations, total and ionized calcium was higher in hypercalcemic men compared to normocalcemic men (2.69 mmol/L vs. 2.36 mmol/L, and 1.46 mmol/L vs. 1.24 mmol/L, respectively). In response, PTH concentrations were significantly lower in hypercalcemic men (1.0 pmol/L vs. 2.6 pmol/L, *p* < 0.001). Average 1,25(OH)_2_D_3_ concentrations were also higher in hypercalcemic men (154 pmol/L vs. 116 pmol/L, *p* = 0.005), while the mean 25(OH)D level was slightly higher as well (54 nmol/L vs. 46 nmol/L, *p* = 0.081). Magnesium (*p* = 0.446), phosphate (*p* = 0.762), sodium (*p* = 0.088), and potassium (*p* = 0.298) concentrations did not differ significantly between groups. Iron levels were lower in hypercalcemic men compared to normocalcemic men (16.3 µmol/L vs. 19.2 µmol/L, *p* = 0.041). Hemoglobin levels were also significantly lower in the hypercalcemic group (9.3 mmol/L vs. 9.9 mmol/L, *p* = 0.012).

### Associations between oil injection and average serum mineral concentrations

**Fig. 2:**
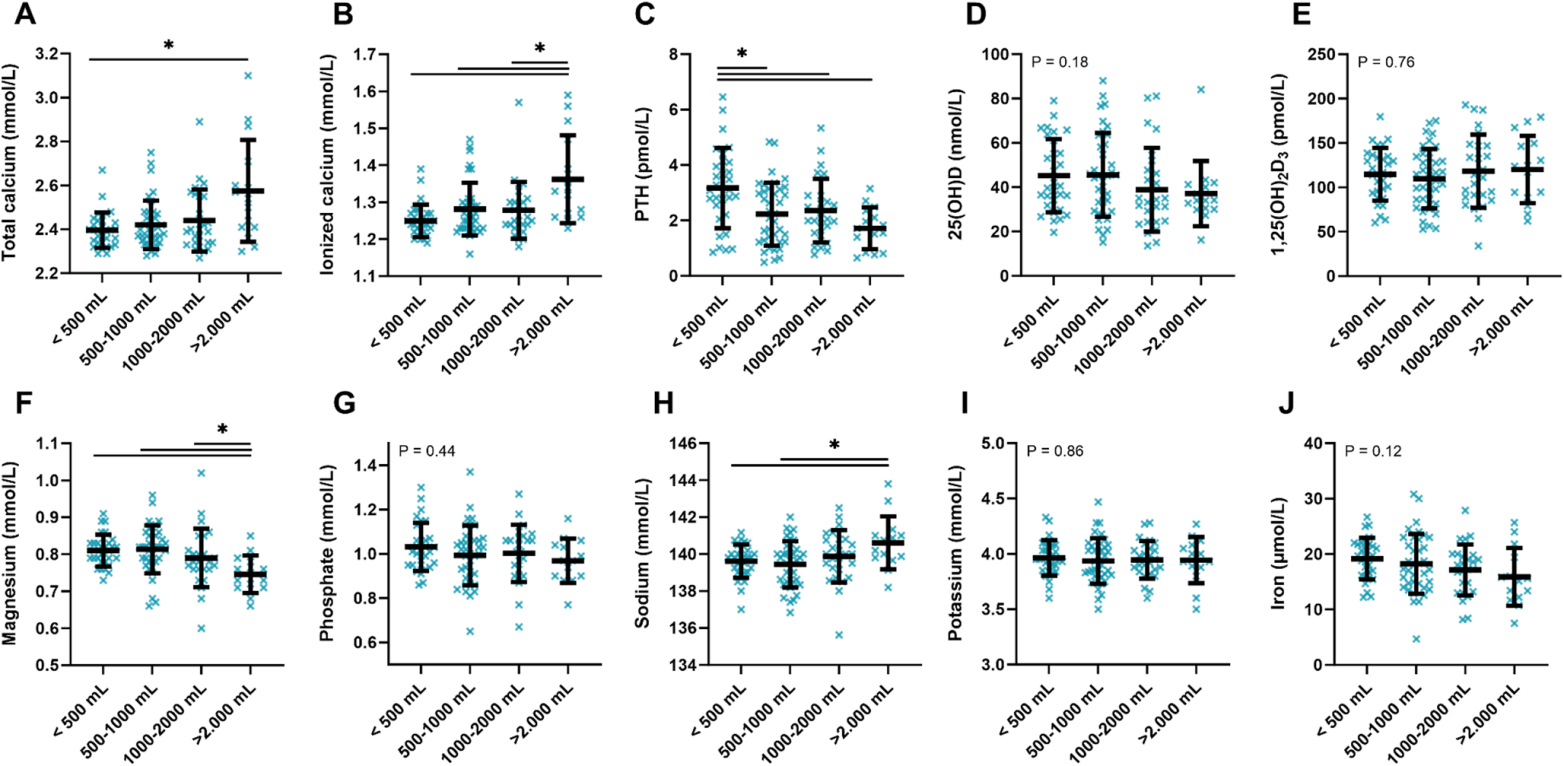
Association between the amount of oil injected and concentrations of serum calcium, PTH, 25(OH)D, 1,25(OH)_2_D_3_, and serum minerals. Serum concentrations of minerals grouped by self-reported amount of oil injected. (**A**) Total calcium, (**B**) Ionized calcium, (**C**) PTH, (**D**) 25(OH)D, (**E**) 1,25(OH)_2_D_3_, (**F**) Magnesium, (**G**) Phosphate, (**H**) Sodium, (**I**) Potassium, and (**J**) Iron. Green crosses represent each individual and black lines represent the mean with standard deviation. P-value for differences between the groups was assessed by a one-way ANOVA test and post-hoc pairwise comparisons adjusted with Dunnett’s test. Abbreviations: PTH: parathyroid hormone; 25(OH)D: 25-hydroxyvitamin D; 1,25(OH)_2_D_3_: 1,25-dihydroxyvitamin D

The severity of granuloma disease was also assessed by stratifying men according to the self-reported amount of oil injected (< 500 mL, 500-1,000 mL, 1,000–2,000 mL, and > 2,000 mL), as shown in Fig. 2. Serum concentrations of calcium and PTH were correlated with the amount of oil injection. Men who had injected > 2,000 mL had higher total calcium (2.57 mmol/L vs. 2.40 mmol/L, *p* = 0.029, Fig. 2A) and ionized calcium (1.36 mmol/L vs. 1.25 mmol/L, *p* < 0.001, Fig. 2B) compared to men who had injected < 500 mL. Accordingly, serum PTH concentrations were lower (1.7 pmol/L vs. 3.2 pmol/L, *p* < 0.001, Fig. 2C), but also when compared to men who had injected 500-1,000 mL (1.7 pmol/L vs. 2.2 pmol/L, *p* = 0.008) and 1,000–2,000 mL (1.7 pmol/L vs. 2.4 pmol/L, *p* = 0.031). While there was a slight trend of lower 25(OH)D, and higher 1,25(OH)_2_D_3_ concentrations with more amount of oil injected, the differences were not significant (*p* = 0.18 and *p* = 0.76, respectively, Fig. 2D and E). However, the men who had injected > 2,000 mL had lower serum magnesium concentrations compared to the other groups, 0.75 mmol/L vs. 0.79 mmol/L, 0.81 mmol/L, and 0.82 mmol/L, respectively (*p* < 0.001, *p* < 0.001, and *p* = 0.042). They also had higher sodium concentrations compared to < 500 mL (141 mmol/L vs. 139 mmol/L, *p* = 0.048, Fig. 2H). No differences were found in phosphate (*p* = 0.44), potassium (*p* = 0.86), and iron concentrations (*p* = 0.12) (Fig. 2G and I, and 2J).

### Changes in total calcium, PTH, 25(OH)D, and 1,25(OH)_2_D_3_

**Fig. 3:**
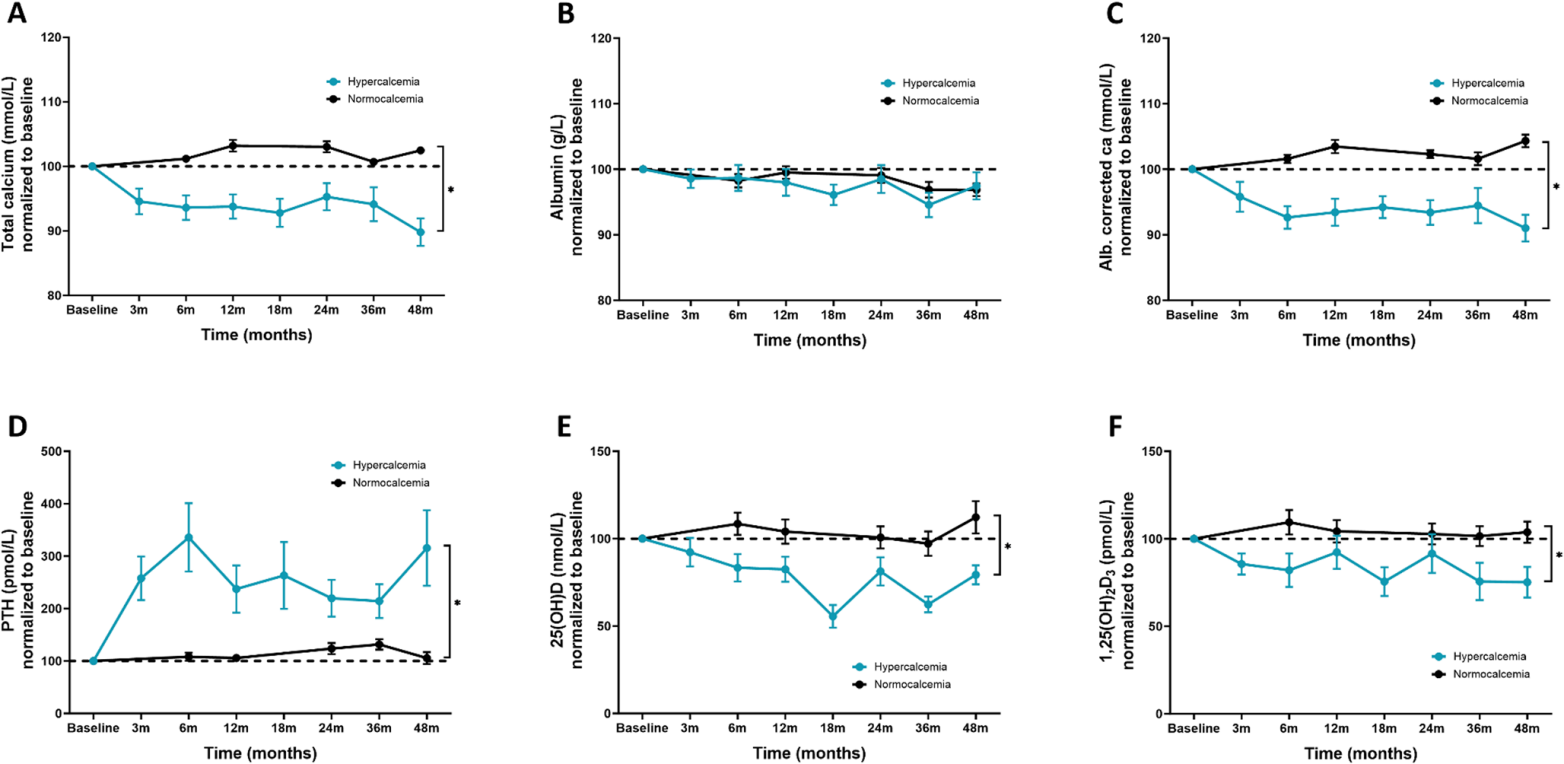
Measured concentrations of total calcium, PTH, 25(OH)D, and 1,25(OH)_2_D_3_ for hypercalcemic and normocalcemic men from baseline with follow-up to 48 months. (**A**-**D**) Normalized plots for serum concentrations of total calcium, PTH, 25(OH)D, and 1,25(OH)_2_D_3_ in patients grouped according to calcium status at baseline. Data from baseline, and after 1, 3, 6, 12, 19, 24, 36, and 48 months are shown. Differences between groups at 48 months were compared by a crude comparison of means (t-test). * p-value < 0.05

When stratified according to baseline calcium concentrations, men with hypercalcemia had a decrease in total calcium over time, while men with normocalcemia remained close to baseline concentrations for up to 48 months (Fig. 3A). Men with hypercalcemia dropped from a baseline concentration of total calcium at 2.69 mmol/L (SD 0.30) to 2.46 mmol/L (SD 0.15) after 48 months, while men with normocalcemia had slight changes from 2.36 mmol/L (SD 0.08) to 2.38 mmol/L (SD 0.10). When normalizing concentrations to baseline, and comparing the groups at 48 months, there was a significant difference (*p* < 0.001) in the values (Fig. 3A). The same changes were observed in albumin corrected calcium (Fig. 3C). The opposite reaction was seen in serum PTH, where men with hypercalcemia had a decrease in PTH from 1.0 pmol/L (SD 0.7) at baseline, to 2.1 pmol/L (SD 2) after 48 months, with a significant difference (*p* < 0.001) when compared to men with normocalcemia (Fig. 3D). Also, concentrations of 25(OH)D showed a decrease in men with hypercalcemia, even though the values were in the normal range at baseline, reflecting the restriction in vitamin D intake and sun exposure (Fig. 3E). 1,25(OH)_2_D_3_ decreased from 154 pmol/L (SD 66) at baseline, to 97 pmol/L (SD 48) in men with hypercalcemia after 48 months (*p* = 0.042) (Fig. 3F).

### Changes in ionized calcium, magnesium, phosphate, sodium, potassium, and iron

**Fig. 4:**
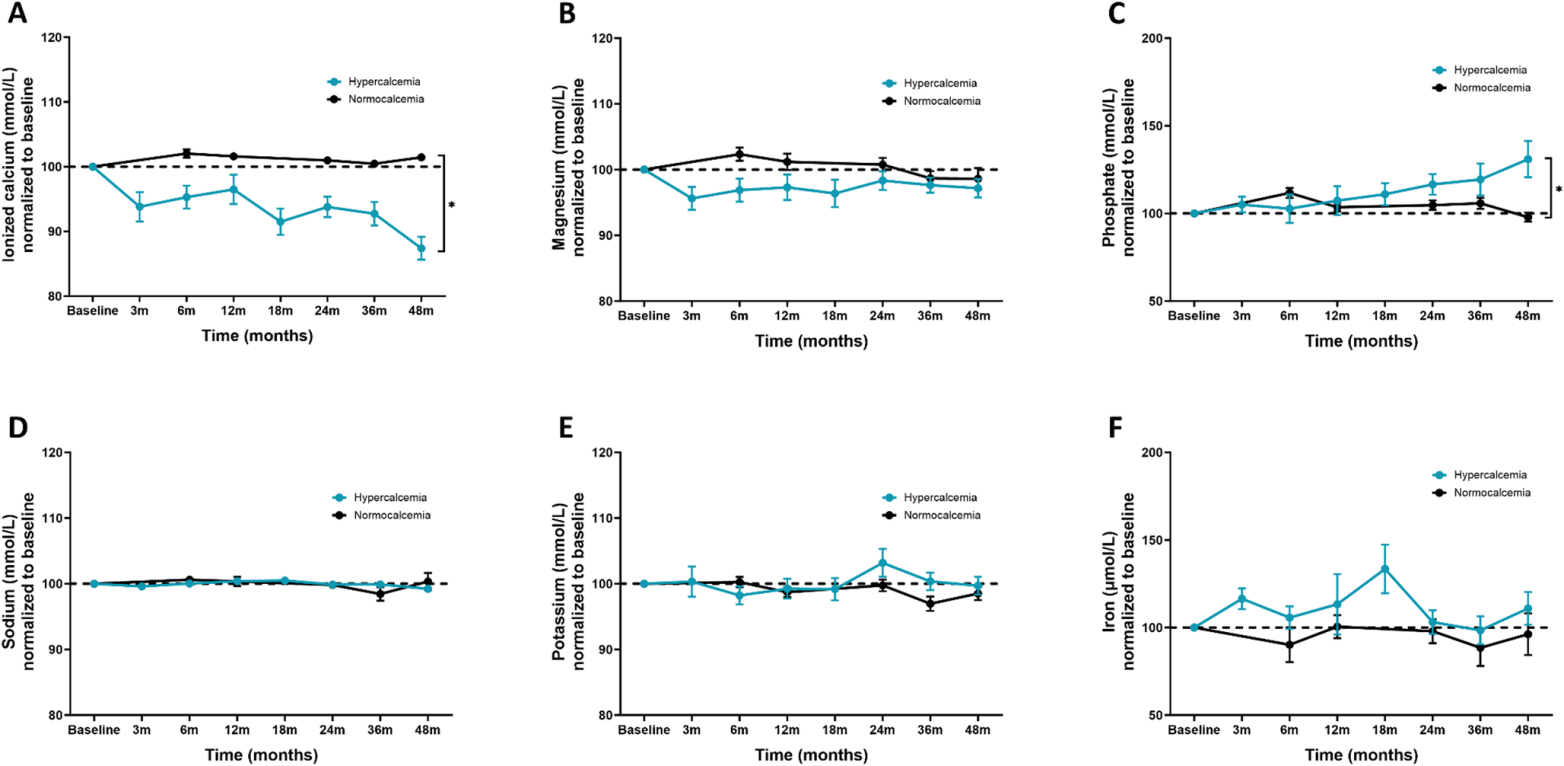
Measured concentrations of ionized calcium, magnesium, phosphate, sodium, potassium, and iron for hypercalcemic and normocalcemic men from baseline with follow-up to 48 months. (**A**-**F**) Normalized plots for serum concentrations of ionized calcium, magnesium, phosphate, sodium, potassium, and iron in patients grouped according to calcium status at baseline. Data from baseline, and after 3, 6, 12, 19, 24, 36, and 48 months are shown. Differences between groups at 48 months were compared by a crude comparison of means (t-test). * p-value < 0.05

Ionized calcium showed a similar trend to total calcium, as men with hypercalcemia dropped and ended on different (*p* < 0.001) normalized values compared to men with normocalcemia (Fig. 4A). Of the 79 men who were normocalcemic, only 5 (6%) developed hypercalcemia (ionized ca. > 1.32 mmol/L) after 36 months, and only 2 (2.5%) after 48 months (data not shown). While men who were normocalcemic at baseline did not experience a change in serum phosphate through the follow-up period, men with hypercalcemia saw a rise from 1.01 mmol/L (SD 0.41) at baseline, to 1.10 mmol/L (SD 0.46) in men with hypercalcemia after 48 months (*p* = 0.042 when compared to men with normocalcemia). Serum iron concentrations had a transient increase to their highest point at 17.1 µmol/L after 18 months in men with hypercalcemia but decreased back to baseline levels, and no changes were seen in ferritin concentrations (data not shown). Concentrations of magnesium (Fig. 4B), sodium (Fig. 4D), and potassium (Fig. 4E) did not change or differ between the two groups over time. Kidney function improved slightly in men with hypercalcemia from a baseline eGFR of 69 mL/min/1.73 m^2^ (SD 21) to 73 mL/min/1.73 m^2^ (SD 21) but with no difference compared to the normocalcemic group for either eGFR or serum creatinine (data not shown).

### Changes in serum minerals after prednisolone treatment

**Fig. 5:**
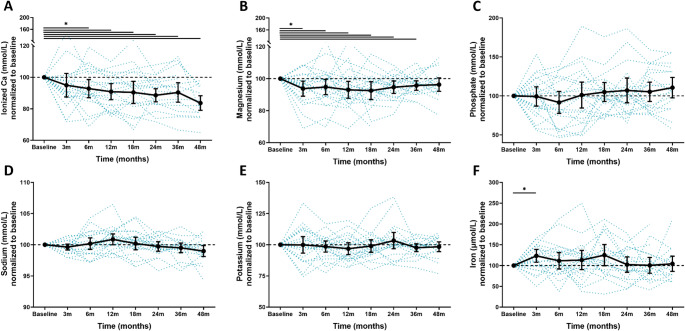
Measured concentrations of ionized calcium, magnesium, phosphate, sodium, potassium, and iron for patients treated with prednisolone from baseline with follow-up to 48 months. (**A**-**F**) Normalized plots for serum concentrations of ionized calcium, magnesium, phosphate, sodium, potassium, and iron in patients who have been treated with prednisolone. Data from baseline, and after 3, 6, 12, 18, 24, 36, and 12 months are shown. Differences between groups were compared by a linear mixed model for repeated measurements and Dunnett’s test to adjust for multiple comparisons. * p-value < 0.05

A subgroup of 21 men were treated with prednisolone at varying lengths during the follow-up. The average dose of treatment was 23 mg/daily (SD 15) at baseline, decreasing to 17.5 mg/daily (SD 11.9) after three months, 15 mg/daily (SD 7.5) after six months, 8.75 mg/daily (SD 5) after twelve months, 5 mg/daily (SD 5) after 18 months and 24 months, before increasing slightly to 7.5 mg/daily (SD 5) after 36 and 48 months. Figure 5 shows serum concentrations of ionized calcium, magnesium, phosphate, sodium, potassium, and iron normalized to baseline for patients treated with prednisolone from baseline and up to 48 months. Serum ionized calcium concentrations gradually dropped in the follow-up period (Fig. 5A). Baseline average calcium ion levels were measured at 1.52 mmol/L (SD 0.20), which decreased to 1.44 mmol/L (SD 0.22) after three months (*p* = 0.240), 1.40 mmol/L (SD 0.16) after six months (*p* = 0.042), and 1.38 mmol/L (SDs 0.13 and 0.15) after 12 and 18 months (*p* = 0.028 and *p* = 0.036, respectively). It further dropped to 1.34 mmol/L (SD 0.13) after 24 months (*p* = 0.011), before normalizing at 1.32 mmol/L (SD 0.14) after 36 months (*p* = 0.035) and ending at 1.27 mmol/L (SD 0.06) after 48 months (*p* < 0.01). Serum magnesium concentrations also followed the same trend (Fig. 5B) starting with baseline average concentration at 0.81 mmol/L (SD 0.11), before dropping to 0.76 (SD 0.11) and 0.77 mmol/L (SD 0.11) after three and six months (*p* = 0.030 and *p* = 0.045). It further dropped to 0.75 mmol/L (SD 0.15) after 18 months (*p* = 0.042), 0.76 mmol/L (SD 0.13) after 24 months (*p* = 0.025), and 0.75 mmol/L (SD 0.09) after 36 months (*p* = 0.027) and ended on 0.77 mmol/L after 48 months (*p* = 0.251). There was a small drop in phosphate concentration after six months, decreasing from baseline average concentration of 1.05 mmol/L (SD 0.26) to 0.87 mmol/L (SD 0.22), but later increased again over time to baseline levels (Fig. 5C). Finally, there was an initial increase in iron concentration, where it rose from baseline average concentrations at 14.9 µmol/L (SD 6.3) to 17.6 µmol/L (SD 6.8) after 6 months (*p* = 0.039) but later normalized close to baseline again over time (Fig. 5F). No significant changes were observed in sodium and potassium concentrations (Fig. 5D and E).

## Discussion

This study shows that granuloma disease can cause alterations in mineral homeostasis although calcium is the most sensitive cation. The severity of the disease evaluated by calcium homeostasis depends on the volume of oil injected and a clear correlation between the amount of oil injected. Men who injected higher amounts of paraffin oil (i.e., > 2.000 mL) exhibited a higher risk of hypercalcemia approx. 12 years later, with a notable compensatory suppression of PTH concentrations. Furthermore, concentrations of 1,25(OH)_2_D_3_ also correlated with the volume of oil injected, indicating a more severe disease with higher extra-renal production of 1,25-dihydroxyvitamin D and thereby elevated serum calcium levels. Furthermore, serum concentrations of magnesium were lower in men who injected > 2,000 mL of paraffin compared to all three groups who had injected a lower amount. Even though serum magnesium concentrations were not under the threshold for hypomagnesemia (< 0.70 mmol/L), the difference of ~ 10% (0.82 mmol/L compared to 0.75 mmol/L) may be of some importance although with no obvious clinical significance [31, 32]. Magnesium plays a crucial role in calcium homeostasis, and its deficiency can contribute to altered calcium regulation including PTH release. Also, iron levels were reduced in correlation with the amount of oil injected, which may indicate a broader disruption in mineral metabolism for instance by regulation of FGF23 that is linked with iron or a direct toxic effect of the paraffin oil on iron absorption or utilization. Sodium levels were also higher, while we have no explanation for this, the inflammatory response might lead to changes in kidney function, aldosterone release, or activity resulting in elevated sodium levels over time.

When the severity of the granuloma disease was stratified according to baseline calcium concentrations, men with manifest hypercalcemia at baseline had different slopes of mineral concentration during the 5 years of follow-up. Hypercalcemic men had a bigger drop in calcium and an increase of PTH as expected, but also lowered activated vitamin D status. However, the men with hypercalcemia experienced an increase in serum phosphate concentrations over time, and a transient increase in iron concentration, although this reached baseline levels again after 24 months. Paradoxically, phosphate levels increase when PTH rises significantly, which may be explained by a decline in GFR and thus lower phosphate excretion despite the increased PTH. Moreover, there may be less FGF23 action either due to lower circulating FGF23 or impaired klotho activity, although we have no data to support this theoretical suggestion. The normocalcemic men did not show any changes in concentrations. This indicates a more complex and potentially unstable mineral balance during the follow-up period the more the disease has progressed. When assessing patients treated medically with prednisolone, there was a lowering of ionized calcium from hypercalcemia to normocalcemia over time, but also showed changes in other serum minerals, e.g., a significant lowering of serum magnesium over time, and a brief lowering of serum phosphate, and increase in serum iron concentrations, before they normalized again close to baseline levels over time.

Injecting large volumes of paraffin oil into skeletal muscles for cosmetic enhancement is not an uncommon practice among certain environments of young men in Denmark, and this behavior frequently leads to severe health complications, including hypercalcemia or normocalcemia where the calcium concentrations still are compensated by hypoparathyroidism. This is a direct result of granuloma formation that may manifest years after the injection. Hypercalcemia can have significant health implications, including renal dysfunction, cardiovascular issues, and neuromuscular problems, which makes managing patients with granuloma disease a complex process. Patients who required prednisolone treatment generally presented with more severe clinical profiles, including marked hypercalcemia, suppressed PTH levels, and a greater burden of symptoms such as pain. This suggests that systemic corticosteroids may be considered in cases with more aggressive or symptomatic disease. No official guideline for the treatment of granuloma disease following cosmetic oil injections exists, although some parallels have been drawn to other granulomatous diseases such as sarcoidosis [33]. Based on our experience, the initial treatment of these patients would be with corticosteroids, starting at 25–30 mg daily in the initial phase, and aiming for lowering the dose as soon as possible. For persistent usage, the treatment regime should be a combination with a different drug such as tacrolimus, that may allow for reductions in the prednisolone dosages. However, the use of corticosteroids such as prednisolone even in small doses is associated with many side effects, the most important being the development of diabetes, hypertension, obesity, susceptibility to infections, and osteoporosis. Prednisolone lowered serum calcium, magnesium, and phosphate briefly, which may be explained by increased renal excretion of magnesium, as corticosteroids enhance the glomerular filtration rate and decrease the tubular reabsorption of magnesium. Furthermore, longer use of prednisolone may impair the absorption of magnesium in the intestines, contributing further to lower serum magnesium levels. Overall, the use of prednisolone can exacerbate hypomagnesemia, especially in individuals who may already be at risk due to granuloma disease affecting mineral homeostasis, therefore, monitoring serum magnesium levels during prednisolone therapy is crucial to manage and mitigate potential deficiencies.

Higher concentrations of phosphate, as observed in men with hypercalcemia in this study, may further exacerbate the risk of vascular calcification although serum phosphate is still within the upper reference. The transient changes in phosphate levels seen in these patients suggest that granuloma disease disrupts not only calcium but also phosphate metabolism, potentially leading to complex imbalances that could affect overall health and disease progression. While our study did not demonstrate direct clinical consequences related to phosphate levels, the transient elevations suggest that granuloma disease may influence phosphate metabolism alongside calcium dysregulation. One autopsy case revealed ectopic calcification in atypical organs such as the heart, raising the possibility of adverse outcomes [34], though a direct causal link to phosphate levels cannot be established based on our data. The observed reduction in serum iron levels among those with higher oil injections could indicate an inflammatory response or impaired absorption due to the chronic inflammatory state induced by granulomas.

This study is based on a large cohort of patients and is the best available evidence for changes in patient biochemistry while we hopefully in the near future can have data from better trial setups, and ideally a randomized controlled trial. While it has several strengths, some limitations to the study must also be mentioned. The lack of a placebo or a formalized control group is a major limitation when assessing effect, but comparisons between normocalcemic and hypercalcemic men are the best available grouping in this regard. A general limitation in this patient group is low attendance at follow-up appointments, however, this study managed to achieve a relatively high follow-up rate compared to similar research. Despite this, the low attendance at follow-up appointments could still introduce bias, as those who attend may differ systematically from those who do not, potentially skewing the results. Finally, the generalizability of the findings may be limited by the specific demographic and geographic characteristics of the cohort, which may not be representative of the broader population of patients who undergo cosmetic oil injections. The use of different assays for measuring serum (including 1,25-dihydroxyvitamin D) across multiple laboratories, may introduce variability in the results. Another limitation is the lack of systematic assessment of dietary intake, which may have influenced serum iron concentrations. Patients with hypercalcemia were advised to avoid sun exposure and limit dietary sources of calcium and vitamin D, which could have inadvertently reduced their intake of iron-rich foods, contributing to the observed decrease in serum iron levels. Given the limited treatment options and the current gaps in knowledge about effective management strategies, these findings are important, and despite the above-mentioned limitations this study underscores the necessity for comprehensive monitoring of not only calcium, but also magnesium, phosphate, and iron levels, to better manage the systemic effects of the disease and optimize patient outcomes.

In conclusion, this study showed that men who had injected higher amounts of paraffin oil were more likely to have hypercalcemia, lower magnesium, and higher sodium concentrations. Additionally, more advanced disease was associated with greater changes in serum minerals, even in those treated with prednisolone. While these findings are notable, they do not necessarily indicate underlying pathology. To ensure the best treatment for granuloma disease, we emphasize the importance of continuous monitoring and follow-up, including regular assessment of serum minerals such as calcium, magnesium, phosphate, and iron.

## Data Availability

The data that support the findings of this study are available from the corresponding author, M.B.J., upon reasonable request.
